# The role of assistive technology in supporting medication adherence in practice: what are the outcomes and is there evidence for efficacy?

**Published:** 2012-06-15

**Authors:** Manuela Schuette, Nicola Franklin

**Affiliations:** London Borough of Lambeth, UK; London Borough of Lambeth, UK

**Keywords:** assistive technology, medication adherence, outcomes, evidence base, practical application

## Abstract

Medication adherence has a cumulative effect on people’s health and wellbeing and reduces the reliance on health and social care services. This paper reviews the London Borough of Lambeth’s assistive technology service, which is occupational therapy led and includes the use of electronic medication dispensers as part of innovative intervention in support of medication adherence.

The paper gives an appraisal of the key-factors in the successful implementation of medication adherence aids in social care settings and is supported by observational records that originate in practice and by a review of the current evidence base for the use of assistive technologies in medicines management. Key factors include interdisciplinary working as well as having needs-led and outcome focussed services.

In the current economic climate there is increased pressure on local government to deliver services more efficiently and cost-effectively. The paper examines several case studies, which demonstrate how the use of assistive technology can support medication adherence, resulting in cost savings and cost avoidances. A guiding framework with practical recommendations for best practice in implementing medication compliance aids in social care settings is then offered.

There is a need for health and social care workers and others to engage with medication adherence more widely. There is a large and growing new variety of strategies, interventions, technologies and services available which have the potential to aid, facilitate and motivate clients to adhere to their prescribed medications. The paper gives an overview of their practical application and outcomes.

